# Differential interactions between Notch and ID factors control neurogenesis by modulating Hes factor autoregulation

**DOI:** 10.1242/dev.152520

**Published:** 2017-10-01

**Authors:** Marcelo Boareto, Dagmar Iber, Verdon Taylor

**Affiliations:** 1 Department of Biosystems Science and Engineering (D-BSSE), ETH Zurich, Mattenstrasse 26, 4058 Basel, Switzerland; 2 Swiss Institute of Bioinformatics, Mattenstrasse 26, 4058 Basel, Switzerland; 3 Department of Biomedicine, University of Basel, Mattenstrasse 28, 4058 Basel, Switzerland

**Keywords:** Notch signaling, Id transcription factors, Neural stem cells, Neurogenesis, Computational biology

## Abstract

During embryonic and adult neurogenesis, neural stem cells (NSCs) generate the correct number and types of neurons in a temporospatial fashion. Control of NSC activity and fate is crucial for brain formation and homeostasis. Neurogenesis in the embryonic and adult brain differ considerably, but Notch signaling and inhibitor of DNA-binding (ID) factors are pivotal in both. Notch and ID factors regulate NSC maintenance; however, it has been difficult to evaluate how these pathways potentially interact. Here, we combined mathematical modeling with analysis of single-cell transcriptomic data to elucidate unforeseen interactions between the Notch and ID factor pathways. During brain development, Notch signaling dominates and directly regulates Id4 expression, preventing other ID factors from inducing NSC quiescence. Conversely, during adult neurogenesis, Notch signaling and Id2/3 regulate neurogenesis in a complementary manner and ID factors can induce NSC maintenance and quiescence in the absence of Notch. Our analyses unveil key molecular interactions underlying NSC maintenance and mechanistic differences between embryonic and adult neurogenesis. Similar Notch and ID factor interactions may be crucial in other stem cell systems.

## Abstract (precis)

**Summary:** Computational analysis of transcriptome data from neural stem cells reveals key differences in the synergistic interactions between Notch and inhibitor of DNA-binding factors during embryonic and adult neurogenesis.

## INTRODUCTION

**Fig. 1.**
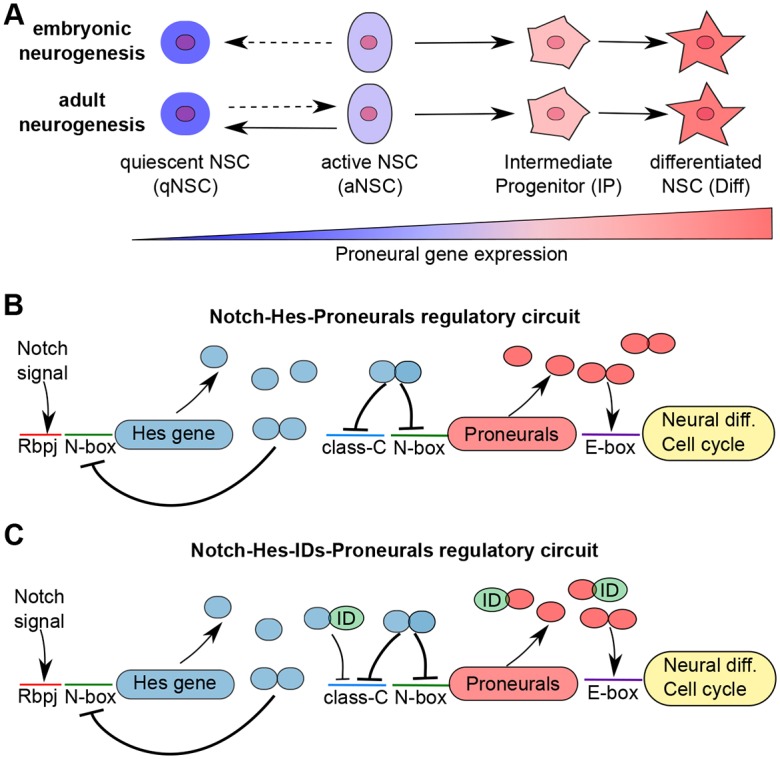
**The NSC differentiation processes in the embryonic and adult brain and its regulatory network.** (A) NSC fate in the embryonic and adult brain is dependent on the levels of proneural transcription factor expression. During embryonic neurogenesis, the majority of the NSCs are in a mitotically active state (aNSC) while a few will enter quiescence (qNSC) and remain inactive until adulthood. In the adult neurogenic niches, most NSCs are mitotically inactive (qNSC) and rarely transit to the mitotically active, neurogenic state (aNSC). In aNSCs, low levels of proneural activity drive cell cycle progression but is insufficient to induce differentiation. In the absence of proneural transcription factor activity, NSCs are quiescent (qNSC) and high proneural transcription factor activity drives neural differentiation (Diff). (B) The Notch-Hes-Proneural transcription factor interaction network. Notch signaling through the DNA-binding protein Rbpj activates expression of Hes genes. Hes protein homodimers repress proneural gene expression, including *Ascl1* and *Neurog2* via N-box and class-C sites, and their own expression by binding to N-box sites in their promotor regions. Proneural transcription factors activate cell cycle progression and differentiation via E-box sites. (C) The current known Notch-Hes-IDs-proneural interactions. IDs form heterodimers with Hes transcription factors, which are unable to bind to N-box sites but can bind to class-C sites, although with lower efficiency than Hes homodimers. IDs also form heterodimers with proneural factors that are unable to activate the differentiation and cell cycle progression genes.

Neurogenesis is the production of neurons from neural stem cells (NSCs). The correct balance between NSC proliferation and differentiation is essential for embryonic formation of the brain and to confer regenerative capacities in the adult brain (Doe, 2008). Any deviation from the regulated neurogenic program can lead to drastic problems during development, including microcephaly and cognitive impairment. During embryonic development of the central nervous system, NSCs divide frequently and produce neurons either directly or via a committed intermediate progenitor (IP) cell (Fig. 1A). In the peak neurogenic period, a few NSCs exit the cell cycle and become quiescent (qNSCs) (Furutachi et al., 2013, 2015; Fuentealba et al., 2015). qNSCs are only reactivated in the adult neurogenic niches. In the adult brain, NSCs remain and neurogenesis is active in two defined regions: the ventricular-subventricular zone (V-SVZ) of the lateral ventricle wall; and the dentate gyrus of the hippocampus (Doetsch et al., 1999; Doetsch, 2003; Spalding et al., 2013; Ernst et al., 2014; Fuentealba et al., 2015; Furutachi et al., 2015). In the adult brain the majority of the NSCs are mitotically inactive (qNSC) and infrequently enter cell cycle, becoming active NSCs (aNSCs) to generate neurons before returning to quiescence or differentiating into glial cells (Fig. 1A) (Lois et al., 1996; Kirschenbaum et al., 1999; Encinas et al., 2011; Ihrie and Alvarez-Buylla, 2011; Shook et al., 2012; Giachino et al., 2014). Thus, although embryonic and adult neurogenesis share some similarities, there are also fundamental differences and stem cell quiescence is one of them. Currently, it is not known why NSCs of the adult brain remain quiescent and the mechanisms that control the transition of NSC to activation are also unclear. However, the balance between activity and quiescence is crucial not only to maintain the NSC pool for later neuron production and regeneration but also to prevent overproliferation and tumor formation (Lugert et al., 2010; Silva-Vargas et al., 2016). Thus, understanding the molecular mechanism that regulates maintenance and differentiation of NSCs is not only of theoretical interest but crucial for understanding disease mechanism and developing new therapeutic strategies (Lie et al., 2004; Lazarov et al., 2010).

The processes of NSC maintenance and differentiation are controlled by a core regulatory network of basic helix-loop-helix (bHLH) transcription factors (Lee, 1997; Ross et al., 2003; Heng and Guillemot, 2013; Imayoshi and Kageyama, 2014b). Members of the bHLH family have two conserved functional domains: a basic region for DNA binding and a helix-loop-helix (HLH) region for dimerization. These transcript factors can act as repressors or activators of gene expression. The hairy and enhancer of split (Hes) proteins Hes1 and Hes5 are central repressors of NSC differentiation during brain development (Ohtsuka et al., 1999; Kageyama et al., 2007, 2008), while bHLH factors including Ascl1 and Neurog2 are activators of neural differentiation and thus referred to as proneural factors (Wilkinson et al., 2013; Imayoshi and Kageyama, 2014b). Hes proteins in conjunction with TLE factors repress gene expression by binding to N-box and class-C sites in the promoters of target genes. Proneural factors activate gene expression by binding to E-box consensus sequences in the promoters of their targets (Fig. 1B) (Imayoshi and Kageyama, 2014b). Furthermore, the binding affinity of proneural factors to E-boxes can be enhanced by the formation of heterodimers with other members of the bHLH family: the E-proteins Tcf4 and Tcf3 (Massari and Murre, 2000; Bohrer et al., 2015).

During brain development, Notch signaling activates Hes gene expression, which in turn inhibits NSC differentiation by repressing proneural genes, including *Ascl1* and *Neurog2* (Lee, 1997; Heng and Guillemot, 2013). In addition, Hes proteins repress expression of their own genes, counteracting Notch and leading to oscillations in their expression (Fig. 1B) (Hirata et al., 2002). This dynamic Hes gene activity has been suggested to result in low-level expression of proneural factors, including Ascl1, and this low expression drives cell cycle progression but is not sufficient to induce NSC differentiation (Castro et al., 2011; Imayoshi et al., 2013; Andersen et al., 2014). In contrast, high and sustained expression of Hes proteins drives complete repression of *Ascl1*, leading to cell cycle exit and NSC entry into a quiescent state (Baek et al., 2006; Castro et al., 2011; Imayoshi et al., 2013; Andersen et al., 2014). NSCs in the adult brain niches are predominantly quiescent, a state not observed frequently in the developing brain. How regulation of the Hes-proneural gene axis is differentially controlled in NSCs during development and in the adult brain is unknown. However, previous observations suggest that different levels of proneural activity in the NSCs lead to three possible output states in the NSCs: NSC quiescence, proliferation and differentiation when proneural activity is absent, intermediate/low and high, respectively (Fig. 1A).

In the adult brain, NSCs quiescence has been linked to the expression of inhibitor of DNA-binding factors (IDs) (Nam and Benezra, 2009). IDs also have a HLH domain, which enables the formation of heterodimers with other bHLH factors, but lack the basic domain and for this reason cannot efficiently bind to DNA (Tzeng, 2003; Heng and Guillemot, 2013). Therefore, IDs act as inhibitors of the activity of bHLH factors. Experimentally, IDs have been shown to form dimers with Hes proteins and these heterodimers are unable to bind to the N-box-binding motifs in the Hes promoter and thus relieve Hes auto-repression (Bai et al., 2007). Interestingly, Hes-ID heterodimers can still repress target genes, including *Ascl1*, via class-C binding sites, albeit with lower efficiency than Hes homodimers (Bai et al., 2007). Thus, IDs are able to segregate auto-repressive and downstream target gene repression functions of Hes factors. In addition, IDs also form ineffective heterodimers with proneural factors, including Ascl1, reducing their potential to drive differentiation by blocking their binding to E-boxes in target genes (Imayoshi and Kageyama, 2014b). Hence, IDs potentially regulate neurogenesis at multiple levels, including enhancing Hes expression and blockage of proneural factor activity (Fig. 1C).

Owing to the complex and reciprocal interplay between Notch-Hes and IDs, it has been challenging to access the consequences of their interactions experimentally and their respective roles in the control of NSC activity. As a first step to address this problem, we developed a specific theoretical framework that takes into account the interactions between Notch, IDs and the members of the bHLH family of transcriptional factors. Our theoretical framework is in line with previous models of Hes (Lewis, 2003; Monk, 2003; Novák and Tyson, 2008; Wang et al., 2011; Pfeuty, 2015) and explicitly incorporates Notch-mediated activation of Hes gene expression, Hes-mediated repression of proneural expression, Hes auto-repression and homodimer formation. In order to recapitulate the different effects of IDs, we incorporated the possibility of Hes-ID and proneural-ID heterodimer formation into the model. We explored computationally the properties of this gene regulatory network and the conditions required to obtain NSC quiescence, maintenance of activated NSCs and differentiation. Once we had established a robust model that fulfilled these criteria, we challenged and validated our predictions by analyzing the gene expression of NSCs at the single-cell level. Finally, by evaluating the differences in the single-cell expression profile of adult and embryonic NSCs, we uncovered key differences between embryonic and adult neurogenesis.

## RESULTS

### Notch signaling alone cannot completely repress proneural activity and drive NSC quiescence

The balance between Notch signal activity and proneural factor expression is pivotal in the regulation of NSC activity and neurogenic differentiation. Proneural factors, including Ascl1, are important for neuronal differentiation but at lower transient levels also induce NSC cell cycle entry. Complete repression of Hes expression is a prerequisite for proneural gene expression to levels that induce progenitor cell commitment and differentiation. By contrast, proneural gene expression is completely repressed in qNSCs and is necessary for NSCs to exit cell cycle (Castro et al., 2011; Imayoshi et al., 2013; Andersen et al., 2014).

**Fig. 2.**
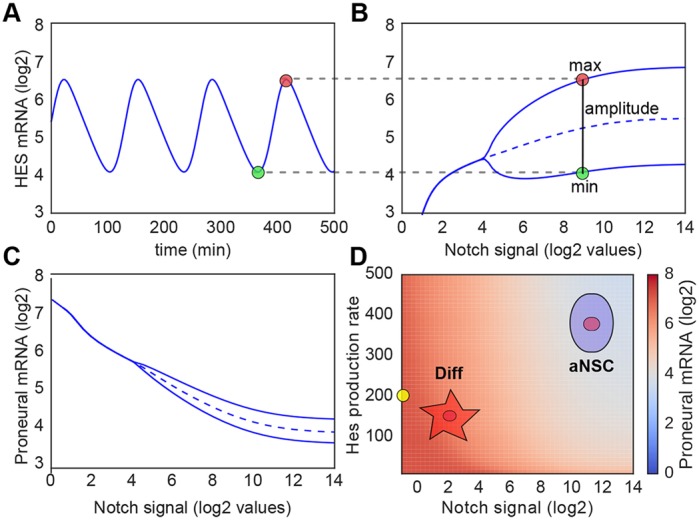
**Notch-Hes-Proneural regulatory module can regulate NSC differentiation but cannot drive NSCs into quiescence.** (A) Hes mRNA levels oscillate with a periodicity of ∼140 min. (B) A minimum activation of gene expression is required for oscillatory behavior, which is induced in response to a large range of Notch signal activation. Upper and lower curves represent the maximum and minimum expression levels of Hes mRNA during the oscillations (as indicated by the red and green circles, respectively). Dashed curve represents the temporal average expression of Hes mRNA. The mean expression level of Hes mRNA does not change dramatically in the oscillatory region of expression, irrespective of the levels of Notch signal activity. (C) Expression level of proneural genes as a function of Notch signaling activity. Proneural gene expression becomes oscillatory at the same level of Notch signal due to the oscillatory expression of Hes factors. (D) Predictions of proneural gene expression and NSC fate at different levels of Notch signal and Hes mRNA production rate. Changes in Hes basal production rate have little effect on proneural gene expression (blue to red heat map) at any given level of Notch signal. Low Notch signal activity leads to high proneural expression (red) and this results in NSC differentiation (Diff). In contrast, high Notch activity leads to low-intermediate levels of proneural expression (light blue) leading to active, proliferative NSCs (aNSC). Production rates are in mRNA/min and the yellow circle indicates the standard value determined experimentally and used in all simulations (see Material and Methods).

Therefore, we initially studied the dynamics of the Notch/Hes regulatory module in NSCs in the absence of any extra factor (Fig. 1B). To be consistent with previous theoretical and experimental evidence (Hirata et al., 2002; Monk, 2003; Wang et al., 2011), we adjusted our model such that Hes gene expression oscillates with a periodicity of 2-3 h in the presence of a Notch signal (see Materials and Methods; Fig. 2A,B). We then evaluated mathematically the effect of Notch activity on the levels of proneural gene expression. In agreement with experimental data, our model recapitulated that increasing Notch signaling decreases proneural gene expression (Fig. 2C). However, Notch signaling alone was unable to completely suppress proneural gene expression or even reduce it to the levels necessary for cell cycle exit (NSC quiescence) (Fig. 2C). We evaluated whether complete repression of proneural gene activity could be achieved by increasing the basal production rate of Hes and maximal levels of Hes by increasing Notch activity (Fig. 2D). Surprisingly, an increase in Hes production had little effect on the levels of proneural gene expression. Moreover, changes in Notch/Hes signaling leads to binary fates: high Notch leads to low-intermediate proneural activity that induces proliferation and active NSCs; low Notch leads to high proneural activity that results in differentiation (Fig. 2D). Therefore, our computational results suggest that, in the absence of any extra factor, Notch signaling cannot completely repress proneural activity and thereby induce NSC quiescence. Interestingly, this situation is seen during embryonic development where most NSCs of the developing brain are mitotically active.

### Hes-negative feedback, and not oscillations, leads to the accumulation of the minimal proneural activity required for NSC proliferation

As quiescence is the major state of NSC in the adult brain, we investigated the conditions required for a complete repression of proneural gene expression. It has been suggested that the oscillatory expression of Hes leads to low levels of proneural factor expression that enable and drive NSC proliferation while being insufficient for differentiation (Kageyama et al., 2009; Imayoshi et al., 2013; Imayoshi and Kageyama, 2014a). In order to investigate the impact of oscillations on proneural expression, we explored the regions of the parameter space where Hes expression no longer oscillates (Fig. S1). Rapid Hes protein degradation, long intronic delay and Hes auto-repression have all been shown to be required for oscillatory Hes expression (Bai et al., 2007; Yoshiura et al., 2007; Takashima et al., 2011). Analytical studies of Hes oscillations have identified intronic delay and protein degradation as key parameters that modulate Hes dynamics (Ay et al., 2013, 2014; Wang et al., 2014). Therefore, we explored how changes in these parameters would affect the expression level of the downstream proneural genes.

**Fig. 3.**
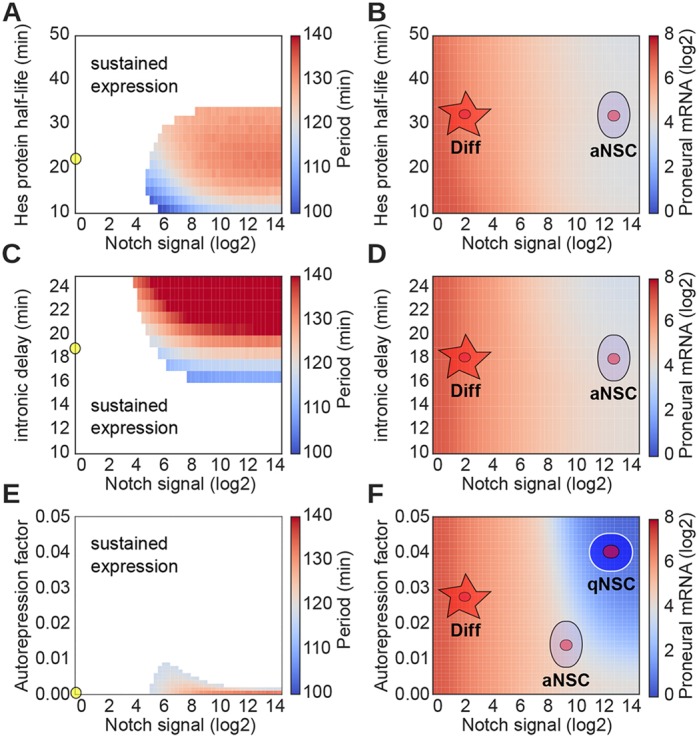
**Release of Hes auto-repression drives complete repression of proneural expression.** (A) Period of oscillations of Hes expression for different values of Notch signal and Hes protein half-life. White area indicates the region where Hes does not oscillate (sustained expression). Yellow circle indicates the standard value used in all simulations. (B) Proneural expression levels for different values of Notch signal and Hes protein half-life. Low Notch activity leads to NSC differentiation (Diff), whereas high Notch activity maintains NSCs active/proliferative (aNSC). Changes in Hes protein degradation have little effect on proneural expression. (C) Period of oscillations of Hes expression and (D) proneural expression levels for different values of Notch signal and intronic delay. Low Notch activity leads to NSC differentiation (Diff), whereas high Notch activity maintains NSCs active/proliferative (aNSC). Changes in intronic delay have little effect on proneural expression. (E) Period of oscillations of Hes expression and (F) proneural expression levels for different values of Notch signal and Hes auto-repression. Release of Hes auto-repression, together with high Notch activity, can drive complete repression of proneural activity, leading to NSC quiescence (qNCS).

We initially evaluated the effects of changing Hes protein half-life on the oscillatory behavior of Hes gene expression (Fig. 3A). Increasing Hes half-life substantially above the experimentally determined 22 min resulted in loss of oscillations and sustained Hes mRNA expression levels (Fig. 3A). Similarly, reducing Hes protein half-life shortened the periodicity of the oscillations (Fig. 3A). These findings are consistent with experimental evidence suggesting that both increases or decreases in Hes protein degradation rate stops or dampens Hes mRNA oscillations, and validates our mathematical framework (Yoshiura et al., 2007; Harima et al., 2014). Interestingly, however, we found that the average levels of proneural mRNA expression do not change with changes in Hes protein degradation rate and does not lead to a complete block in proneural gene levels (Fig. 3B). Hence, regulation of Hes protein degradation in NSCs cannot account for cell cycle exit and quiescence.

Therefore, we examined the effect of intronic delay of transcript maturation rate on oscillatory Hes gene expression, proneural gene activity and NSC fate. It has been shown that Hes genes have three long introns that regulate transcription and splicing of the primary RNA transcript and maturation of the Hes7 mRNA to ∼19 min (Harima et al., 2014). Removal of one or two of the Hes gene introns increases Hes transcript maturation rate and dampens or completely abolishes oscillations in expression (Takashima et al., 2011; Harima et al., 2014). Consistent with this, in our model we observed that the oscillatory behavior of the Hes genes is absent and expression is sustained with rapid transcript production and maturation. Conversely, a longer delay in Hes mRNA maturation leads to an increase in the periodicity of oscillations at most levels of Notch signal activity (Fig. 3C). Our model also predicts that proneural expression is not affected by changes in Hes transcript maturation (production) rate and that it has little or no effect on NSC fate (Fig. 3D). Therefore, we examined the effect of Hes auto-repression on Hes mRNA oscillatory period, proneural gene expression and NSC fate. We considered an auto-repression factor value of 0.0 to represent a complete repression of Hes gene expression by the Hes proteins. Conversely, an auto-repression factor value of 0.05 represents Hes gene repression of 95% with a residual transcription of 5% (Fig. 3E). Under these conditions, a small relief of Hes auto-repression is enough to completely abolish Hes mRNA oscillations and induce sustained Hes gene expression over most of the range of Notch signal (Fig. 3E). This also leads to a substantial decrease in the expression of the proneural genes (Fig. 3F). So our modeling of the Notch network revealed a mechanism by which relatively small reductions in the efficiency of Hes gene auto-repression leads to dramatic changes in proneural gene expression. Furthermore, the modulation of Hes auto-repression predicts a relative transcriptional profile of Notch, Hes and proneural factor genes that determines NSC differentiation, quiescence and active NSC maintenance. Together, these results suggest that the Hes auto-repression restricts the effect of Notch signal on proneural expression, leading to the maintenance of a minimum level of proneural activity even in the presence of high Notch signaling. Therefore, the efficiency of the Hes-negative feedback, and not Hes oscillatory expression per se, is crucial for the small accumulations in proneural expression required for NSC proliferation and differentiation.

### IDs potentiate Notch/Hes activity and drive complete repression of proneural expression

Our mathematical model confirms the crucial importance of regulating Hes auto-repression in order to enable a complete repression of proneural expression and drive NSC quiescence. This is an important finding as it provides a mode for modulating NSC behavior to achieve the transition from NSC quiescence to activation and differentiation. Although IDs have been shown to regulate neurogenesis and NSC activity as well as alter Hes and proneural factor activity, it remains unclear how the combination of IDs, Hes and proneural factor activity achieve the effects observed in genetic manipulation experiments (Baek et al., 2006; Nam and Benezra, 2009).

**Fig. 4.**
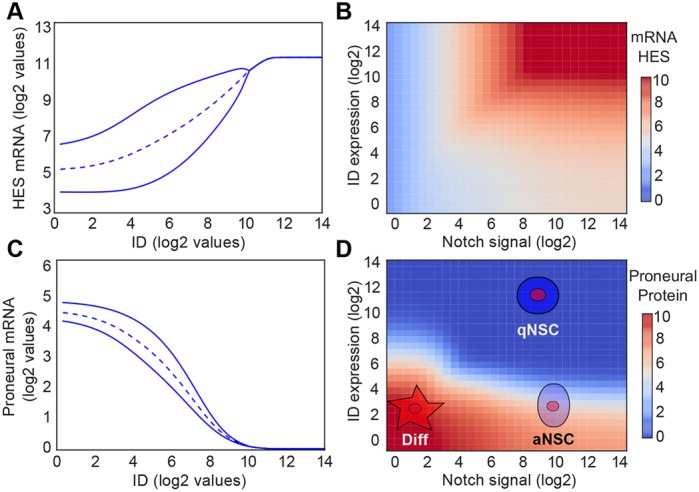
**Combined effect of Notch and IDs can drive NSC differentiation, proliferation and quiescence.** (A) Expression level of Hes mRNA for different levels of ID expression in the presence of constant Notch activity (*I*=500 molecules). (B) Expression level of Hes mRNA relative to different levels of ID expression and Notch activity*.* (C) Expression level of proneural factor mRNAs for different values of ID expression in the presence of constant Notch activity. (D) Levels of proneural factor activity (protein level) for different levels of Notch activity and ID expression. IDs potentiate the effect of Notch signaling by releasing Hes auto-repression and can act in concert with Notch, forming a three-way switch that segregates NSCs into quiescent (qNSC) (high IDs), proliferative/active (aNSC) (low IDs, high Notch/Hes) or differentiated (Diff) (low IDs, low Notch/Hes). The oscillatory region is presented in Fig. S2.

We then incorporated IDs into our theoretical framework and observed that by reducing Hes auto-repression, IDs lead to an increased and sustained expression of Hes genes (Fig. 4A). Thus, Hes expression levels are uncoupled from Notch signal intensity by simply increasing expression of ID proteins, which also results in non-oscillatory expression and high levels of Hes (Fig. 4A). In addition, by evaluating the effects of Notch and IDs on Hes, we found that Hes gene expression only reaches maximum levels in the presence of both Notch signaling and IDs (Fig. 4B). Furthermore, high levels of IDs are able to completely repress proneural gene activity, on the one side by increasing Hes levels to repress transcription either via Hes homodimers or ID-Hes heterodimers, and on the other side by directly interacting with proneural proteins to inhibit target gene activation (Figs 1C and 4C). By combining Notch signals and ID activity, our model then predicts that proneural activity segregates into three expression states that directly translate into different stem cell states: low/absent proneural expression leading to NSC quiescence (qNSCs); low/intermediate proneural levels in the absence of IDs in cells with active Notch signaling, resulting in NSCs being mitotically active (aNSCs); and high proneural activity in the absence of both Notch signaling and IDs, which drives NSC into differentiation (Diff) (Fig. 4D and Fig. S2). This model establishes then clear and sharp boundaries in NSC fate that present a feasible explanation for the experimental data linking IDs and Notch signaling to NSC activation and differentiation.

### Notch and IDs regulate adult neurogenesis in a complementary manner

**Fig. 5.**
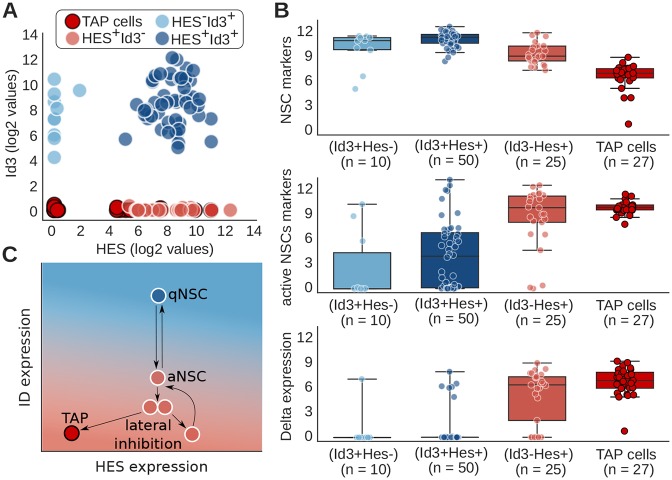
**Expression pattern of adult NSCs at the single-cell level and mechanistic interplay between Notch/Hes and IDs.** (A) Single cells from adult murine brain are represented based on their levels of Hes (Hes1, Hes5) and Id3 (Llorens-Bobadilla et al., 2015). Color code represents three subpopulations of NSCs: Id3+Hes−, Id3+Hes+ and Id3−Hes+; and transient amplified progenitors (TAP) cells. (B) Expression level of NSC markers (Slc1a3, Nr2e1, Sox9, Vcam1), active NSC/progenitor markers (Ascl1, Fos, Egr1, Sox4, Sox11) and Delta ligands (Dll1, Dll3, Dll4) for different populations of NSCs and TAP cells. NSC markers and active NSC/progenitor markers were chosen based on the analysis of the expression profile of adult NSCs (Llorens-Bobadilla et al., 2015). (C) Schematic representation of adult neurogenesis based on the modeling presented in Fig. 4D and on experimental results presented in B. High levels of IDs drive NSC quiescence. By decreasing IDs, the NSC become proliferative and stimulate the expression of the Notch ligand Delta. Notch-Delta lateral inhibition segregates neighboring active NSCs into high and low Notch signal. While the NSC with low Notch differentiates into a TAP cell, the NSCs with high Notch remain proliferative and can go another round of differentiation. Similar results are found using Id2 instead of Id3, or considering all IDs (Id1-Id4) together, and for an alternative choice of NSC and proliferative markers (Figs S5-8).

To test and validate our model and its predictions, we analyzed published expression profiles of single cells isolated from the SVZ of adult mouse forebrain that were classified as NSCs (130 cells; Slc1a3, Nr2e1, Sox9, Vcam1^+^) and transit amplifying progenitors (TAPs, 27 cells; Ascl1, Fos, Egr1, Sox4, Sox11^+^) (Llorens-Bobadilla et al., 2015). We inferred Notch activity from the levels of Hes gene family expression in the data set. Of the four ID genes (*Id1*, *Id2*, *Id3* and *Id4*), *Id2* and *Id3* were expressed in most cells and strongly correlated with NSC marker expression (Figs S3 and S4). Most NSCs (85 out 130) expressed either Hes or Id3 mRNA, and were clearly divided into three subpopulations: Id3^+^Hes^+^ (50 cells), Id3^+^Hes^−^ (10 cells) and Id3^−^Hes^+^ cells (25 cells) (Fig. 5A).

We then compared the expression levels of NSC markers (Slc1a3, Nr2e1, Sox9, Vcam1) (Llorens-Bobadilla et al., 2015) and active NSC/progenitor markers (Ascl1, Fos, Egr1, Sox4, Sox11) (Llorens-Bobadilla et al., 2015) by these progenitor subpopulations. As expected, TAPs (Hes^low^ID^low^) expressed relatively lower levels of the NSC markers than Id3^+^Hes^−^, Id3^+^Hes^+^ and Id3^−^Hes^+^ NSCs (Fig. 5A,B). Interestingly, Id3^+^Hes^+^ and Id3^+^Hes^−^ NSCs expressed low levels of active NSC/progenitor markers, while Id3^−^Hes^+^ cells had high levels of active NSC/progenitor markers. As TAPs are mitotically active and Id3^+^ NSCs do not express genes associated with an active mitotic state, these findings indicate that Id3^+^ cells are mostly quiescent (blue in Fig. 5A,B), whereas most Id3^−^Hes^+^ cells also express markers of proliferation and TAPs (red in Fig. 5B). We come to similar conclusions when we consider the expression of *Id2* in place of *Id3*, or even when considering the expression of all Id genes together (Figs S5 and S6), and when we use alternative NSC and proliferation markers (Figs S7 and S8). The analysis of these experimental data suggest that IDs are sufficient to drive NSC quiescence, which lends direct support to our theoretical model indicating that ID expression and not Notch signaling itself is crucial for NSC quiescence (Fig. 5C) (Nam and Benezra, 2009). Or, put another way, ID expression represses adult NSCs from entering the mitotically active state by blocking proneural gene expression and activity. Mechanistically, our results suggest that downregulation of IDs by qNSCs releases the complete repression of proneural activity induced by sustained high-level Hes expression, and promotes cell cycle entry, at least in part, by enabling proneural factor expression. Our data analysis also indicates that active NSCs express the Notch ligand Delta (Fig. 5B). Notch-Delta signaling leads to lateral inhibition between neighboring cells, segregating them into two populations: receiver cells with high Notch/Hes and low Delta expression; and sender cells with low Notch/Hes and higher Delta expression. Sender cells further differentiate into TAPs while receiver cells remain as active NSCs and can go through another round of proliferation (Fig. 5C). According to our model, NSC differentiation could be controlled by IDs even in the absence of Notch signaling. By lowering the levels of IDs in qNSCs, they can become active/proliferative. A further decrease in the levels of IDs would then lead to differentiation and ultimately to depletion of the NSC population. This is consistent with experimental evidence showing that inhibition of Notch signaling in the adult niche leads to the loss of stem cell population and highlights the crucial role of Notch signaling in controlling NSC maintenance and differentiation (Chapouton et al., 2010; Imayoshi et al., 2010; Basak et al., 2012; Kawaguchi et al., 2013).

### Differences between ID-Notch regulation of embryonic and adult neurogenesis

**Fig. 6.**
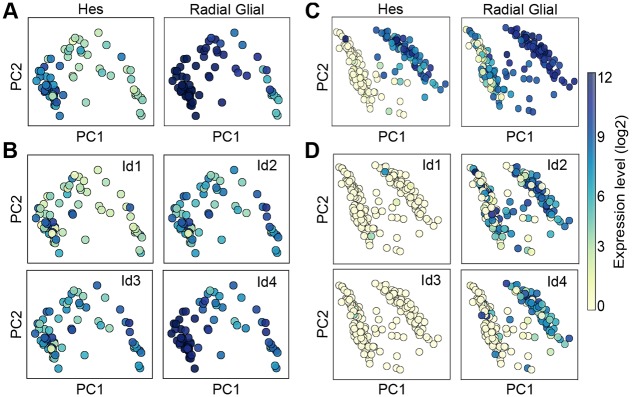
**Hes and IDs expression profile in mouse and human NSCs during embryonic brain development.** (A) PCA representation of single cells from the murine embryo (Kawaguchi et al., 2008). Color represents the expression levels of Hes genes (*Hes1*, *Hes5*) and radial glia (embryonic NSC) markers (*Slc1a3*, *Pax6*, *Sox2*, *Pdgfd*, *Gli3*). (B) Color represents the expression level of Id1-Id4 genes (log2 scale). (C) PCA representation of single cells from the ventricular zone of the human embryo (Pollen et al., 2015). Color represents the expression levels of Hes genes (*Hes1*, *Hes5*) and radial glia markers (*Slc1a3*, *Pax6*, *Sox2*, *Pdgfd*, *Gli3*). (D) Color represents the expression level of Id1-Id4 genes (log2 scale).

Our modeling results show a feasible mechanism by which Notch and IDs collaborate to regulate neurogenesis in a complementary manner. However, during embryonic stages of brain development, most NSCs are mitotically active although they express IDs (Yun et al., 2004). Therefore, we addressed the differences in Notch/Hes and ID interactions in the embryo and adult NSCs, and the reason why Notch-Hes-ID induce quiescence of NSCs in the adult V-SVZ but not during embryonic development. We analyzed publically available expression datasets of single embryonic progenitor cells in the ventricular zone of both murine and human brain (Kawaguchi et al., 2008; Pollen et al., 2015). We found that the cells could be divided into two populations: proliferative NSCs that have high levels of both Hes and radial glial markers (embryonic NSCs; Slc1a3, Pax6, Sox2, Pdgfd and Gli3), and basal intermediate progenitors that express low levels of these radial glial markers and high levels of intermediate progenitors markers (Tbr2, Elavl4, Neurog1, Neurod1, Neurod4, Ppp1r17, Penk) (Fig. 6A,C and Fig. S9). We then evaluated the expression profile of IDs (Id1-4) in these different cells. We found that *Id2* and *Id4* were expressed by a significant fraction of cells and that, in contrast to the situation in adult NSCs, *Id4* but not *Id2* expression overlapped with the expression of Hes genes and radial glia markers (Fig. 6B,D).

The function of Id4 has been identified as a paradigm shift compared with Id1-3 in different tissues during development and in cancer (Patel et al., 2015). *Id4* but not *Id1*, *Id2* and *Id3*, is a target of Notch/Rbpj signaling (Li et al., 2012). Consistent with this, we observed an overlap in expression between *Id4* and Hes genes in embryonic progenitors (Fig. 6). In addition, it has been suggested that Id4 can inhibit the function of other IDs (Sharma et al., 2015). This suggests that Notch regulates the expression of both Hes and Id4 in embryonic NSCs, and that Id4 blocks the inhibitory function of Id1-3. Thus, the predominance of Id4 over Id1-3 during embryonic neurogenesis is a key difference between embryonic and adult NSCs. This suggests that the expression of Id4 induced by Notch signaling prevents Id1-3-modulated repression of Hes autoregulation, thus preventing the sustained high level expression of Hes genes that is required to block proneural gene expression and drive cell cycle exit. In addition, the inhibitory effect of Id4 on the other ID proteins also blocks Id1-3-mediated inhibition of the proliferation. Consistent with this mechanism, neural progenitor cells in *Id4* mutant mice show prolonged G1-S transition during brain development (Yun et al., 2004). Moreover, *Id4*-mutant neural progenitor cells also show precocious differentiation, suggesting that Id4 also plays a role in regulating NSC differentiation, likely via sequestration of proneural factors (Yun et al., 2004).

## DISCUSSION

The regulation of stem cell fate is highly complex. In the nervous system, an ever-increasing number of factors that can change NSC activity and fate are being uncovered. Signaling pathways downstream of many of these factors have either been shown to converge, synergize or even counteract each other. Notch signaling is a central regulator of NSC fate and plays key roles in regulating maintenance, proliferation and differentiation (Nyfeler et al., 2005; Ehm et al., 2010; Imayoshi et al., 2010; Lugert et al., 2010; Basak et al., 2012). The best known mode of Notch activity is to suppress expression of the proneural genes through expression of Hes factors (Lutolf et al., 2002). Hence, deletion of the core DNA binding component of the Notch pathway, Rbpj, or the effectors Hes1 and Hes5, leads to NSC activation and precocious differentiation (Ehm et al., 2010; Imayoshi et al., 2010; Lugert et al., 2010; Basak et al., 2012). Similarly, ID proteins control NSC maintenance and activation in the developing nervous system and control NSC quiescence and fate in the adult brain (Nam and Benezra, 2009). IDs are downstream components of the TGFβ pathway but share bHLH transcriptional regulators of the Hes and proneural family as common targets with the Notch pathway (Viñals et al., 2004; Yun et al., 2004; Bai et al., 2007; Nam and Benezra, 2009). IDs form heterodimers with bHLH factors, which produces inactive complexes with most partners. However, heterodimers of Hes and IDs retain some activity, particularly at the promoters of proneural factor targets (Bai et al., 2007).

It has been a major challenge to understand how Notch signaling controls fate. Until recently Notch signaling was considered to be a molecular switch, activated by lateral signaling between neighboring cells. More recently, it was found that, rather than being a switch, Notch signaling is highly dynamic; in addition, in-built oscillatory expression of the Notch target genes *Hes1* and *Hes5* is crucial to Notch function. The dynamic expression of Hes proteins projects onto the proneural genes whose expression oscillates out of phase with the Hes genes. During embryonic neurogenesis, Notch signaling and a dynamic expression of Hes genes and proneural genes is the predominant mechanism regulating NSC maintenance and differentiation; both show a salt-and-pepper pattern in NSCs of the VZ. This dynamic Notch activity coincides with most NSCs being mitotically active, whereas neurogenic differentiation is blocked by repressing proneural gene expression to low levels and preventing accumulation of these neurogenic factors to levels sufficient for differentiation. The relationship between Hes oscillations and NSC proliferation is intriguing and raises the possibility that NSC fate is also based on the dynamic behavior of Hes proteins. Our results suggest that the low proneural factor activity required for NSC proliferation, can be generated independently of Hes oscillations, although oscillations might provide a robust way of keeping proneural factor levels low. However, we do not exclude the possibility that Hes oscillations lead to an alternative mechanism independent of proneural factor expression through which NSC fate can be controlled. Here, we show that this dominance of Notch signaling in embryonic NSCs is due to synergy between Notch signaling and Id4. Id4 expression correlates strongly with Hes gene expression (and Notch activity) in both human and mouse progenitors isolated from the embryonic brain. Furthermore, both Hes gene and *Id4* expression are enriched in NSCs rather than TAPs and more committed cells. These findings parallel data indicating that *Id4* is a transcriptional target of Notch signaling (Li et al., 2012). Therefore, in embryonic NSCs Id4 blocks proneural activity and target gene activation while enabling and supporting Hes auto-repression and maintaining oscillations in Hes gene expression. We propose that this is a key requirement during embryogenesis, as the dynamics in Notch and proneural activity enable a rapid transition of the NSCs to differentiation during brain development. In addition, our model proposes that Id2 and Id3, although expressed by NSCs and committed progenitors during embryonic development are outcompeted for common partners by Id4, preventing the formation of inactive Hes-Id2/3 heterodimers (Sharma et al., 2015).

Conversely, in the adult nervous system, most NSCs are mitotically inactive and, in contrast to embryonic NSCs, Notch signaling does not oscillate and proneural gene expression is absent in most NSCs, except those that activate and enter cell cycle (Imayoshi and Kageyama, 2014a). The mechanism that inhibits the oscillatory expression of the Notch effectors *Hes1* and *Hes5* has been elusive. However, it has been demonstrated that high level Notch signaling and maintained expression of Hes1 are required to induce NSC quiescence and this correlates with ID expression (Hirata et al., 2002; Nam and Benezra, 2009; Imayoshi et al., 2013). How both processes are regulated and how Notch switches from promoting quiescence to blocking differentiation of aNSCs is unclear (Chapouton et al., 2010; Imayoshi et al., 2010; Lugert et al., 2010; Basak et al., 2012). We show computationally for the first time that the synergistic and antagonistic interactions between Hes factors and IDs are central to both of these mechanisms during adult neurogenesis. We modeled computationally different paradigms to test how the oscillatory expression of Hes could be modulated and found that reducing the auto-repression on the Hes promoter was more effective in stabilizing *Hes* gene expression than changing Hes protein stability or even increasing its expression levels. Our results suggest that Id2 and Id3 form ineffective heterodimers with Hes proteins and reduce the auto-repressive feedback on the Hes promoters in qNSCs. This results in increased Hes protein expression and complete repression of the proneural genes. Hence, although Id2/3 can repress proneural factor activity on their target genes, our model indicates that their major role in inducing NSC quiescence is to relieve Hes auto-repression.

In summary, computational modeling has enabled us to reconcile different experimental findings and data and provide a solid hypothesis for how Notch and IDs work together to regulate neurogenesis in the embryonic and adult brain. It is astonishing that the predictions of our model were supported by gene expression data both from mouse and human. Furthermore, our model demonstrates key differences in Notch and ID activity in embryonic and adult NSCs, and provides an explanation for the quiescent state observed in adult NSC. Why adult NSCs are predominantly quiescent although they remain sensitive to Notch signaling is a central question in the adult neurogenesis field. Hence, the use of mathematically modeling and validation by analysis of unbiased sequence data allowed us to unravel a complex cross-regulatory network mechanism that has been difficult to address experimentally. However, as our model now uncovers the multiple potential modes of action of ID proteins in the regulation of Notch activity and NSC fate, it will be important to address these complex Hes-ID interactions and their functions using cell biological and genetic experiments *in vivo*. Because IDs can potentially modulate any bHLH factor, it is expected that these factors play different and even opposite roles in different systems. Therefore, understanding the role of ID interactions with other factors will require development of specific theoretical frameworks. This will allow novel hypotheses of complex biological processes to be developed that can be examined and validated with currently available data or specifically designed experimental approaches.

## MATERIALS AND METHODS

### Theoretical framework

Our model is inspired by a reductionist approach proposed by Julian Lewis in 2003 to study the oscillatory dynamics of Hes/her genes (Lewis, 2003). Lewis introduced a simple model that captures many key features of Hes/Her oscillatory dynamics during somitogenesis and served as a theoretical foundation of many other mathematical models that was further developed to elucidate Hes/Her oscillatory dynamics in different tissues (Lewis, 2003; Monk, 2003; Novák and Tyson, 2008; Wang et al., 2011; Pfeuty, 2015). Therefore, we considered the dynamics of Hes mRNA (*m*) and Hes protein (*p*) to be regulated by auto-inhibition with a transcriptional delay (Eqns 1,2).

As we observe that the expression levels of the Hes genes can reach a maximum of 2^12^ reads per million (RPM) (Fig. 5A). We assumed that one cell expresses a total of 0.5 million mRNA molecules and thus a maximum of 2^11^ (∼2000) Hes transcripts per cell. We used the following model of mRNA regulation:

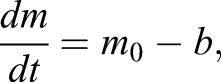
where *m_0_* is the mRNA transcription rate and *b* is the degradation rate. In the equilibrium *dm/dt=*0, therefore, *m=m_0_/b*. The Hes mRNA half-life has been measured to be in the order of 20 min, resulting in *b* being ∼1/20=0.05 per minute. Hence, from the equation *m_0_=bm*, in order to obtain an expression level (*m*) of 2000 transcripts per cell, the production rate *m_0_* would be (*m_0_=*0.05×2000) ∼100 mRNAs per minute. Our choice of an *m_0_*=200 mRNA per minute sets the absolute upper limit for Hes mRNA gene expression. The effective transcription rate in Eqn 1 depends not only on the transcription rate (*m_0_*), but also on the positive regulation of the Notch signal [H^+^(I)] and negative regulation via the auto-repression [H^−^(p_2_[t-t_i_])]. Because the values of the Hill functions are always <1, this leads to a decrease in the effective transcript production rate. In the presence of typical levels of Notch signal and Hes auto-repression, this leads to an effective transcription rate of a few dozens transcripts per minute.

In order to incorporate the effect of IDs, we expanded this model by incorporating the formation of Hes-Hes homo-dimers (*p_2_*) and Hes-ID hetero-dimers (*p_id_*) (Eqns 3,4):
(1)
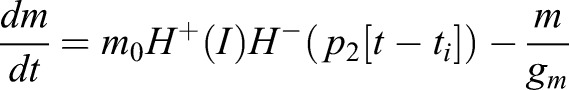

(2)
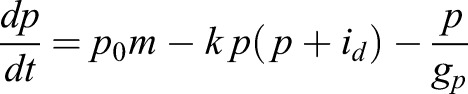

(3)
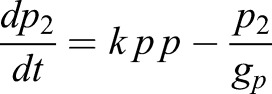

(4)
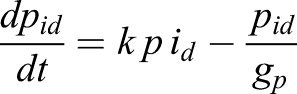
where the positive and negative Hill functions are given, respectively, by:
(5)
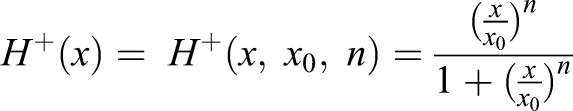

(6)
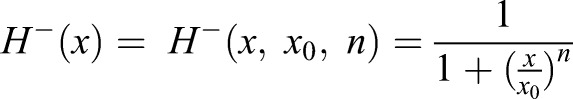
and the parameters *x_0_* and *n* are the Hill factor and Hill coefficient, respectively.

We considered that Hes mRNA is produced by a rate *m_0_* and can be modulated positively by an external input *I* (Notch signal), as represented by a positive Hill function (*H^+^*), and negatively by Hes homo-dimer (*p_2_*), as represented by a negative Hill function (*H^−^*). Similarly, Hes protein is produced by a translation rate of *p_0_*. Based on experimental measurements, we considered that the half-life of Hes mRNA (*g_m_*) and Hes protein (*g_p_*) are 24 and 22 min, respectively (Hirata et al., 2002). For simplicity we assumed that the degradation rate of Hes monomer, Hes-Hes homo-dimer and Hes-ID hetero-dimer are the same (*g_p_*). We also assumed a high affinity between Hes monomers, and between Hes and ID monomers, represented by the variable *k*. The maturation of Hes mRNA has been shown to be delayed by 19 min due to intronic processing (Harima et al., 2014). For simplicity, we considered that this is the only delay involved in the process (*t_i_*=19 min), although extra delays are expected due to transport of mRNA from the nucleus to cytoplasm, protein production and dimer formation. Analytical studies have shown that the oscillatory behavior of Hes genes is highly dependent on the intronic delay and Hill coefficient (cooperativity coefficient) (Bernard et al., 2006). The delay and Hill coefficient have contrasting effects on Hes dynamics: longer delays require lower levels of cooperativity in order to maintain oscillations (Bernard et al., 2006). Therefore, by considering only the intronic delay, a high cooperativity was required to obtain oscillations (*n=5*). Similar dynamics were obtained by considering a delay *t_i_*=25 min and Hill coefficient equal to *n*=4 (Fig. S10). The translation rate (*p_0_*) was chosen so that protein values were in a biological range and oscillations maintained in the order of 2-3 h.

We also took into account the dynamics of a target bHLH gene *a*, which represents a proneural gene such as *Ascl1*. We considered that this gene is activated at a rate of *m_a0_* and degraded a rate of *g_a_*. Experimental evidence suggests that IDs can release Hes gene-mediated auto-repression via N-boxes, but cannot release Hes gene-mediated repression on proneural genes via class-C sites (Bai et al., 2007). Therefore, we considered that this gene is repressed by both Hes-Hes homo-dimers (*p_2_*) and Hes-ID hetero-dimers (*p_id_*), in contrast to Hes genes, which are repressed only by Hes-Hes homo-dimers. We also assumed that Hes-ID heterodimers are less efficient than Hes-Hes homodimers, where **ε** represents the relative strength of repression of Hes-ID when compared with Hes-Hes. We assumed that **ε**=0.5 (twice the concentration of Hes-ID is required to have the same repressive effect as Hes-Hes). A sensitivity analysis evaluating the effect of each parameter of Hes circuit on the levels of both Hes and proneural factors is presented in Fig. S1.
(7)
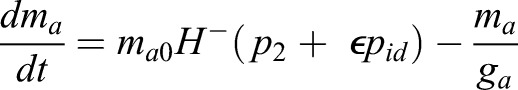

(8)
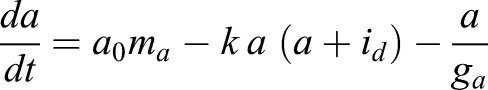

(9)
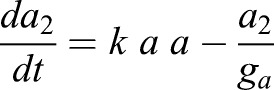

(10)
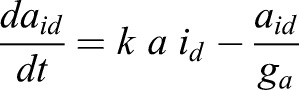
We assumed that the levels of proneural factors, represented by the variables *m_a_* (expression level) and *a_2_* (activity level), controls the fate of the NSCs, irrespective of whether Hes gene dynamics are oscillatory or sustained. Whether the oscillatory expression of Hes genes leads to NSC proliferation via a mechanism besides small accumulations of proneural factor activity remains to be determined. Here, we assumed that the mean high, low/intermediate and low/absent levels of proneural factors drive NSC differentiation, proliferation and quiescence, respectively. It should be noted that our results are qualitative than quantitative in nature.

We considered the amount of IDs available to interact with Hes and proneural factors to be constant. However, ID gene expression can be highly dynamic and has been shown to oscillate in other tissues (William et al., 2007). To discuss the dynamics of IDs and their effects on proneural gene expression, we expanded our model by incorporating one extra equation describing the dynamics of the IDs (supplementary Materials and Methods, Figs S11-13).

To consider the release of Hes gene-mediated auto-repression (Fig. 3E,F), we replaced the negative Hill function in Eqn 1 with a shifted Hill function *H*^*S*^(*x*)=*H*^−^ (*x*)+*f H*^+^ (*x*), where *f* represents the auto-repression factor or fold change of repression and *f*=0 represents a complete repression while *f*=1 represent no repressive effect.

Parameter values used in the simulations are shown in Table 1 unless indicated otherwise. The same Hill factor (*h_0_*) was used in all Hill functions.

**Table 1.**
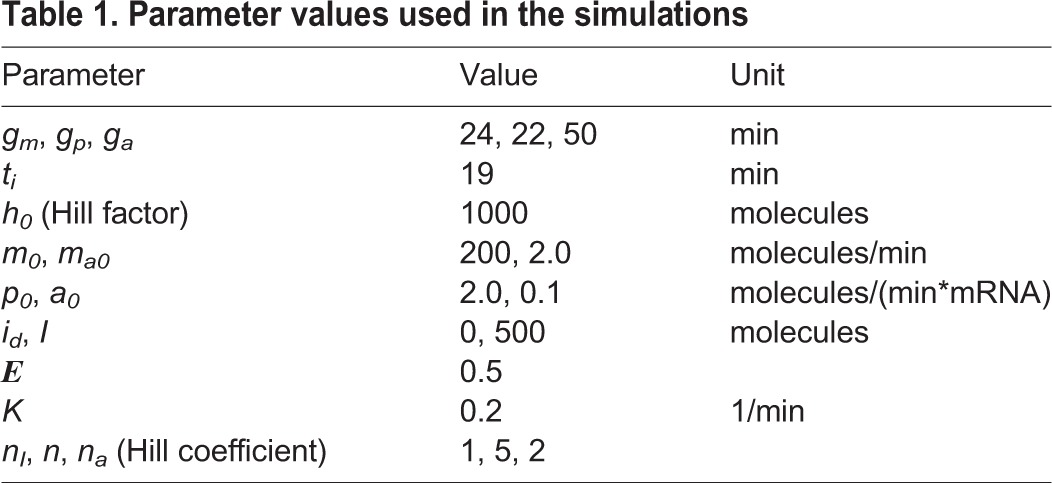
**Parameter values used in the simulations**

### Single cell transcriptomics datasets

We used recently published datasets in order to validate our model predictions. Two datasets were used to evaluate embryonic NSCs. The first dataset consists of cells extracted from the ventricular zone (VZ) and subventricular zone (SVZ) of human embryos at gestation week 16-18 (GW16-18) (Llorens-Bobadilla et al., 2015). By selecting only the cells from the VZ and with more than 1 million reads, we analyzed 179 cells. The second dataset consists of cells from the embryonic murine brain at E14.5 (Kawaguchi et al., 2008; Pollen et al., 2015). We also evaluated the profile of single cells from the adult murine brain between 8 and 12 weeks of age (Llorens-Bobadilla et al., 2015). We selected all the cells annotated as NSCs (total of 130 cells) and as TAP (total of 27 cells). Cells from the mice with ischemia were not used.

Expression levels are represented by the number of reads per million (RPM) in the log2 scale: expression level=log2(RPM+1). Radial glia, NSCs and active NSCs markers were chosen based on the markers suggested by the original manuscript that introduces the database (Llorens-Bobadilla et al., 2015; Pollen et al., 2015). All analysis can be reproduced by following the tutorial source code available on GitHub: http://github.com/mboareto/InterplayNotchID_neurogenesis.
